# Partially Hydrolyzed Guar Gum Intake Supports the Gut Microbiota and Attenuates Inflammation during Influenza H1N1 Virus Infection in Mice

**DOI:** 10.3390/nu15194252

**Published:** 2023-10-03

**Authors:** Takahiro Kato, Satoshi Kamiya, Soshi Narasaki, Ayako Sumii, Yasuo M. Tsutsumi, Kyoka Machida, Kanako Hara, Yuna Izumi-Mishima, Rie Tsutsumi, Hiroshi Sakaue

**Affiliations:** 1 Department of Anesthesiology, Institute of Biomedical Sciences, Hiroshima University Graduate School, Hiroshima 739-8511, Japan; 2 Department of Nutrition and Metabolism, Institute of Biomedical Sciences, Tokushima University Graduate School, Tokushima 770-8501, Japan

**Keywords:** partially hydrolyzed guar gum, H1N1, influenza virus, immunity, butyric acid

## Abstract

Partially hydrolyzed guar gum (PHGG) is a soluble dietary fiber that is effective for defecation control. It influences the gut microbiota, by which it is metabolized to yield short-chain fatty acids (SCFAs), and it was also recently shown to protect against influenza infection in humans. We here investigated the effects of PHGG in a mouse model of influenza H1N1 virus infection. Eight-week-old C57BL/6 mice were fed normal chow with or without PHGG (500 mg/kg per day) for 4 weeks, infected with H1N1 at 10 weeks of age, and analyzed at 12 weeks of age. Administration of PHGG attenuated the decline in body weight induced by H1N1 infection without affecting food intake. It also ameliorated intestinal atrophy and increased the production of SCFAs including acetic acid, propionic acid, and butyric acid in the cecum, thereby preventing the inhibitory effect of H1N1 infection on SCFA production. The H1N1-induced increases in the serum concentrations of inflammatory cytokines including interferon-γ and interleukin-6 and anti-inflammatory cytokine such as interleukin-10 were all inhibited by PHGG intake. In addition, PHGG administration attenuated inflammatory gene expression in the lung and promoted both natural killer cell activity and regulatory T-cell differentiation in the spleen. Our findings suggest that the consumption of PHGG may improve the gut environment and thereby limit the inflammatory response to H1N1 infection. They may thus provide the basis for novel dietary intervention strategies to suppress the excessive inflammation associated with virus infection.

## 1. Introduction

Infectious diseases such as influenza and the coronavirus disease 2019 (COVID-19) are one of the important health problems in modern society. Decreased immune system function leads to infectious diseases, weakened resistance to infection, repeat infection, or severe symptoms. The severity of these diseases range from mild in >80% of confirmed cases to serious, with the latter being characterized by respiratory limitation requiring intensive care and being largely restricted to elderly individuals or those with comorbidities [1,2]. Good nutrition might therefore be expected to have a positive effect on infectious diseases. Alternatively, these individuals are more likely to be infected with pathogens that during normal times, do not typically cause infection. The relation between immunity and nutrition has long been established and is reflected by the use of immunological measures for the assessment of nutritional status [3,4]. More than one-fifth of deaths among adults in 2017 were the result of a poor diet, with healthy eating being found to improve the immune system and immune-related metabolism [5].

Nutritional status including both obesity and malnutrition affects the outcome of infectious disease through multiple mechanisms. Malnutrition is associated with a decline in protein stores and influences the immune system. Low circulating levels of prealbumin, a marker of malnutrition, have been shown to predict the progression to respiratory failure and mechanical ventilation in patients with COVID-19 [6]. On the other hand, the condition of being overweight or obese leads to overproduction of the proinflammatory cytokine interleukin (IL)–6 and reduces the cytotoxicity of natural killer (NK) cells [7].

Symptoms of respiratory infections are not limited to the respiratory tract, and gastrointestinal symptoms such as diarrhea and cramps are also common sequelae [8]. Microbial communities are present in niches in much of the human body, including the gut, skin, lungs, and other mucous membrane surfaces [9,10]. The gut is the most densely and diversely colonized organ [11]. Gut bacteria synthesize vitamins, facilitate nutrient metabolism, and mediate immunomodulation [12]. However, the diversity and abundance of healthy gut microbiota could be disrupted by variety of factors, resulting in dysbiosis with pathogenic growth and depletion of commensal bacteria [13]. Such dysbiosis has been related to various diseases including asthma, diabetes, cancer, and Parkinson’s disease [14,15,16,17].

Partially hydrolyzed guar gum (PHGG) is a water-soluble dietary fiber prepared from guar gum and is thought to have high potential as a prebiotic [13]. PHGG has been demonstrated to ameliorate symptoms associated with both constipation- and diarrhea-predominant forms of irritable bowel syndrome [18] and to provide beneficial effects in the treatment of cholera, small intestinal bacterial overgrowth, pediatric functional gastrointestinal disorders, and metabolic syndrome-related abnormalities such as aberrant lipid and glucose metabolism [19,20,21,22]. PHGG passes in the upper gastrointestinal tract in an undigested form but is fermented by colonic bacteria, resulting in the production of short-chain fatty acids (SCFAs), especially butyrate [23]. Indeed, it was shown to increase the number of butyrate-producing bacteria in the intestine [24]. PHGG treatment was also recently found to alter the composition of the human gut microbiota as well as to increase the abundance of metabolites such as butyrate, acetate, and various amino acids [25]. The beneficial health effects of PHGG are thus likely mediated through alterations to the gut microbiota and SCFA production. A randomized controlled trial recently found that PHGG supplementation contributes to the prevention of influenza infection [26] although the mechanism of this action remains unknown. We examined the possible prebiotic effects of PHGG on inflammation and the gut microbiota in a murine model of influenza infectious disease associated with increased intestinal permeability.

## 2. Materials and Methods

### 2.1. Animals and Diet

All animal experiments were approved by the institutional animal care and use committee at the University of Tokushima Graduate School (Tokushima, Japan; approval number: 2020-96). Male C57BL/6 mice were obtained from SLC Japan (Shizuoka, Japan) and were maintained under a specific pathogen-free condition at 25° ± 3 °C with controlled humidity and on a 12-h-light and 12-h-dark cycle. They were fed normal chow (MF; Oriental Yeast, Tokyo, Japan), which was supplemented with PHGG (Nestle Japan, Tokyo, Japan), at 500 mg/kg daily or with phosphate-buffered saline (PBS) as a control for 4 weeks beginning at 8 weeks of age. They were also provided with water ad libitum. All mice were monitored daily for body weight and for food intake, the latter of which was assessed by measuring the difference between the food offered and that remaining after 24 h. The dose of PHGG was calculated to be 2000 mg/day, with the difference in metabolism between mice and humans being taking into account based on the fact that 5–10 g/day of PHGG has been shown to improve diarrhea and constipation in humans. In addition, PHGG was administered for a total of two weeks, one week as a period sufficient to alter the intestinal environment and another one week to examine the effects of influenza infection.

### 2.2. H1N1 Virus Preparation

The influenza A virus H1N1 was obtained from Sino Biological (Shanghai, China). It was propagated in Madin–Darby canine kidney (MDCK) cells, and its titer was determined with a plaque assay as previously described [27]. MDCK cells were maintained in 6 cm dishes containing Dulbecco’s modified Eagle’s medium (DMEM) supplemented with 10% fetal bovine serum (FBS). For quantitation of virus titer, H1N1 (0.2 mL of a 1000-fold dilution of culture supernatant) was added to MDCK cell monolayers in six-well plates and incubated for 90 min, after which the cells were overlaid with 4 mL of 1% methylcellulose and maintained under 5% CO_2_ at 37 °C for 3 days. The number of plaques in each well was counted after staining with crystal violet, and the viral titer was calculated as plaque-forming units (PFU) per milliliter. Mice were anesthetized with pentverbital and infected intranasally at 10 weeks of age by dropping 20 μL of influenza virus suspension (2× the median lethal dose [LD_50_]) in PBS containing 0.1% bovine serum albumin into one nostril.

### 2.3. Histology

The small intestine was dissected, rinsed with PBS, fixed with 10% formalin, dehydrated, and embedded in paraffin. Sections were then cut at a thickness of 5 μm, stained with hematoxylin-eosin, and examined via light microscopy (Keyence BZ-X800 microscope, Osaka, Japan) at 40× magnification. The length of ileal villi in stained sections was determined with the use of Image J software (NIH, Bethesda, MD, USA).

### 2.4. Gene Expression Analysis

Total RNA was extracted from the ileum or lung with the use of Trizol (Invitrogen, Carlsbad, CA, USA), and portions of the RNA (1 μg) were subjected to reverse transcription (RT) with the use of a Takara Prime Script II 1st Strand cDNA Synthesis Kit (Takara Bio, Shiga, Japan). The resulting cDNA (50 ng) was then subjected to real-time polymerase chain reaction (PCR) analysis in a volume of 10 µL containing primers (Table 1) and Fast SYBR Green Master Mix (Applied Biosystems, Carlsbad, CA, USA) and with the use of a StepOnePlus device (Applied Biosystems). All samples were analyzed in duplicate in a single 96-well reaction plate. Data were analyzed with the ΔCt method, and the expression of target genes was normalized through use of the GAPDH gene.

### 2.5. Flow Cytometry

The spleen was isolated from mice at 12 weeks of age. Dissociated splenocytes were stimulated for 4 h at 37 °C with phorbol 12-myristate 13-acetate (Calbiochem-Novabiochem, San Diego, CA, USA), ionomycin (Sigma-Aldrich, St. Louis, MO, USA), and brefeldin A (Biolegend, San Diego, CA, USA) each at a concentration of 50 ng/mL in RPMI 1640 medium (Gibco BRL, Carlsbad, CA, USA) supplemented with 10% FBS. The cells were then washed, incubated on ice with Fc Block (Bay Bioscience, Hyogo, Japan), and stained with phycoerythrin-conjugated antibodies to CD4 (Bay Bioscience). The cells were then permeabilized with intracellular staining permeabilization and wash buffer (Life Technologies, Eugene, OR, USA) and stained with Alexa Fluor 488-conjugated antibodies to Foxp3 (BD Biosciences, Franklin Lakes, NJ, USA) and Alexa Fluor 647-conjugated antibodies to IL-10 (BD Biosciences) before flow cytometric analysis. The NK cell activity of mouse splenocytes was also assessed with flow cytometry [28]. The cells were washed three times with RPMI 1640 medium, resuspended at a density of 2.5 × 10^5^/mL, and added at 1 × 10^6^ cells per well (40:1 ratio, respectively) in a total volume of 200 μL per well to a 96-well round-bottomed plate (Corning Inc., Corning, NY, USA). The plate was centrifuged (30× *g*, 1 min) and then incubated for 4 h at 37 °C under a humidified atmosphere of 5% CO_2_. Fifteen minutes before the end of the incubation, 20 μL of propidium iodide (Sigma-Aldrich) at 0.5 mg/mL in PBS was added to each well to label dead cells.

All cell samples were subjected to flow cytometric analysis with an Attune NxT Acoustic Focusing Cytometer and Attune NxT software (Life Technologies Japan, Tokyo, Japan). NK cell activity was expressed as the percentage effector cell-specific lysis.

### 2.6. Measurement of IgA and Cytokine Levels

Blood samples were collected from the heart and were centrifuged at 1200× *g* for 10 min at 4 °C to isolate serum. The concentration of immunoglobulin A (IgA) in serum as well as in homogenates of the spleen and in intestinal lavage fluid was determined with an enzyme-linked immunosorbent assay (ELISA) kit (Glory Science, Changhua, Taiwan). Cytokine levels in human serum were determined using mouse IL-6, IL-1b, IL-10, and IFN-g ELISA kits (Abcam, Waltham, MA, USA) according to the manufacturer’s protocol. Absorbance for all assays was measured with a TECAN Sunrise plate reader (Tecan Japan, Kanagawa, Japan).

### 2.7. Determination of Microbiota via 16S rRNA Gene Sequencing

The murine fecal samples were collected from the cecum after a 4-week treatment with PHGG, placed in tubes, and weighed. Bacterial DNA was extracted using the NucleoSpin DNA Stool kit (TaKaRa Bio Inc., Shiga, Japan) according to the manufacturer’s protocol. The 16S ribosomal RNA gene sequencing was performed by Bioengineering Lab Co., Ltd. (Kanagawa, Japan). Briefly, the bacterial 16S ribosomal RNA gene amplicon sequence library was prepared. To analyze the V4 region, the first PCR was performed with Bakt_341F and Bakt_805R primers, and the second amplification was conducted with the index primers. Equimolar amounts of purified DNA amplicons were further amplified on a GridlON X5 platform (Oxford nanopore Technologies, Oxford, UK) for paired-end sequencing (2 × 300 bp) according to the standard protocols presented by Bioengineering Lab Co., Ltd. (Kanagawa, Japan). QIIME version 2.0 (2019.4) was used with the default parameter values for sequence denoising and with the DADA2 method for chimera checking. The Operational Taxonomic Units (OTUs) table was clustered with a 97% similarity cutoff based on the open-reference approach using UCLUST.

### 2.8. Measurements of SCFAs in Cecum with GC-MS

Cecal content (30 mg) was homogenized in 0.5 mL of PBS and then mixed with 300 µL of 2-ethylbutyric acid (20 µg/mL), subjected to ultrasonic treatment, and incubated with shaking for 15 min at 4 °C before centrifugation at 500× *g* for 20 min at 4 °C. A portion of the resulting supernatant (100 µL) was mixed well with 100 µL of diethyl ether and 10 µL of 5 M HCl, and the mixture was centrifuged at 10,000× *g* for 3 min at 4 °C. A portion (80 µL) of the upper layer was added to a tube containing Na_2_SO_4_, and this diethyl ether extraction process was repeated an additional two times with separate portions of the original sample supernatant. A portion (60 µL) of the combined extract was added to a vial insert containing 20 µL of *N*-methyl-*N*-*tert*-butyl dimethylsilyltrifluoroacetamide (Sigma-Aldrich) with the use of a dedicated syringe, and the mixture was shaken gently and maintained in the dark at room temperature for ~12 h to allow derivatization. Derivatized SCFAs were separated via gas chromatography (GC) with the use of a 7890B system (Agilent Technologies, Santa Clara, CA, USA) equipped with a fused silica capillary (122-5532G, Agilent Technologies). Portions (1 µL) of samples were injected in a 10:1 split mode and with a flow rate of helium gas of 1.0 mL/min. The oven temperature was set to 40 °C for 2 min initially, increased first to 170 °C at 15 °C/min and then to 300 °C at 90 °C/min, and finally held at 300 °C for 5 min. Separated SCFA derivatives were detected via mass spectrometry (MS) with a 5977A system (Agilent Technologies) in the selected ion monitoring mode (117 *m*/*z* for acetic acid, 131 *m*/*z* for propionic acid, 145 *m*/*z* for butyric acid, and 173 *m*/*z* for 2-ethylbutyric acid). Quantification was performed by comparing the ratio of the SCFA peak area to the internal standard (2-ethylbutyric acid) peak area for samples to that for reference standards with the use of Agilent MassHunter Qualitative Analysis software 07.00.

### 2.9. Statistical Analysis

Data are presented as mean ± standard deviation (SD). Multiple comparisons were performed with the Kruskal-Wallis test or two-way analysis of variance (ANOVA). The Tukey-Kramer test was applied to identify pairs that showed a significant difference (*p* < 0.05). All statistical analyses and graph generation were performed with the use of PRISM 7 software (GraphPad Software, San Diego, CA, USA).

## 3. Results

### 3.1. Effects of PHGG on Body and Tissue Weights and Food Intake

**Figure 1:**
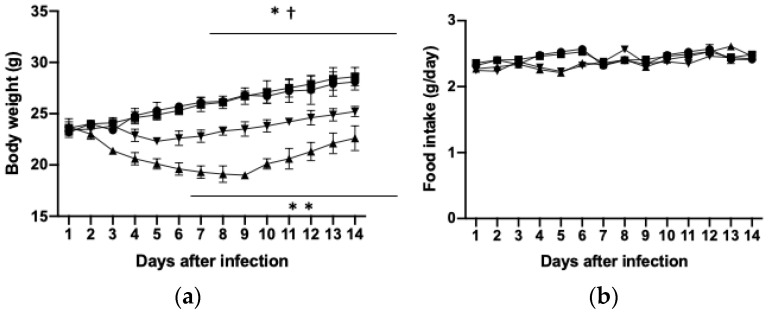
Effects of H1N1 infection and PHGG administration on body weight and food intake. Eight-week-old mice were fed a diet supplemented with PHGG or with PBS as a control for 4 weeks and were infected with H1N1 influenza virus at 10 weeks of age. Body weight (**a**) and food intake (**b**) were measured daily for 2 weeks after virus infection. Circle—control; Square—PHGG; Triangle—H1N1; Inverted triangle—H1N1+PHGG. Data are means ± SD (*n* = 6 mice per group) and were analyzed with the Kruskal-Wallis test for multiple comparisons followed by the Tukey-Kramer test for pairwise comparisons. * *p* < 0.05, control versus H1N1 + PHGG; † *p* < 0.05, H1N1 + PHGG versus H1N1; ** *p* < 0.01, control versus H1N1.

We examined the effects of PHGG administration in a mouse model of H1N1 influenza virus infection. The mice were fed a diet supplemented with PHGG or with PBS as a control for 4 weeks, starting at 8 weeks of age, and were infected with H1N1 at 10 weeks of age. Body weight of mice declined for ~1 week after H1N1 infection and then started to recover, whereas this loss of body weight induced by the virus was significantly ameliorated by PHGG administration (Figure 1a). Determination of tissue weights at 2 weeks after H1N1 infection revealed that the weights of white adipose tissue (visceral and subcutaneous) and skeletal muscle (soleus, extensor digitorum longus, and gastrocnemius) were reduced while those of the liver and spleen were increased in infected mice and that these changes were attenuated by PHGG treatment (Table 2). Neither H1N1 infection nor PHGG administration affected daily food intake (Figure 1b).

### 3.2. PHGG Protected the Gut Environment

**Figure 2:**
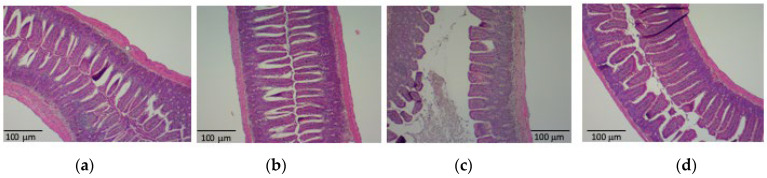

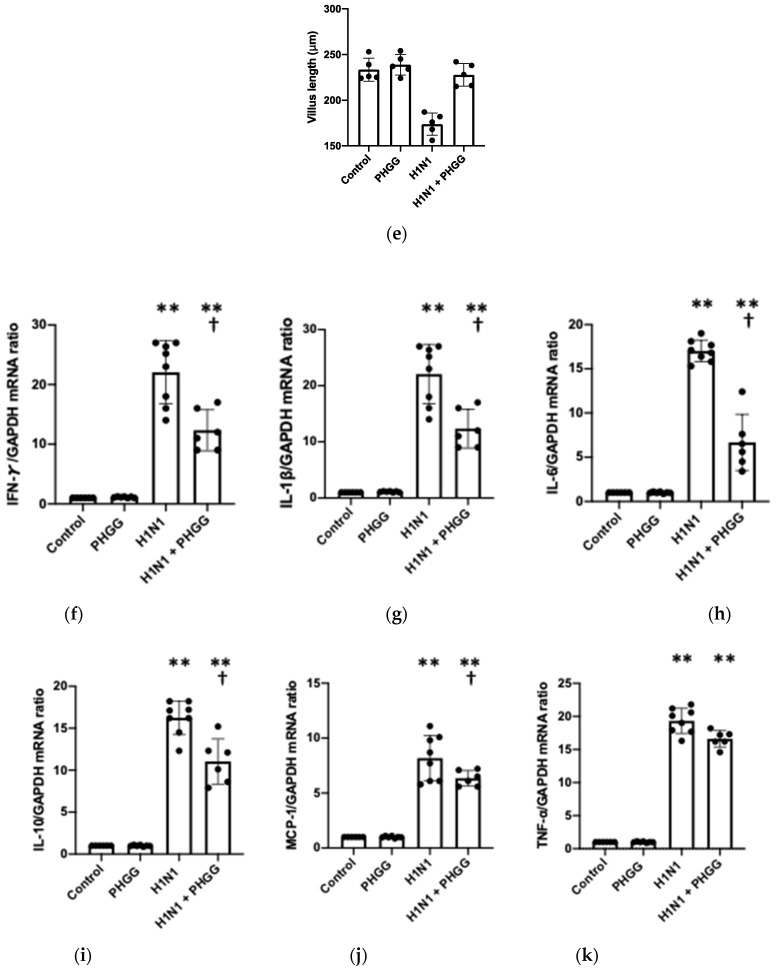
Effects of H1N1 infection and PHGG administration on the histology of villi and cytokine gene expression in the ileum of mice. (**a**–**d**) Hematoxylin-eosin staining of ileal sections from control mice (**a**), PHGG-treated mice (**b**), H1N1-infected mice (**c**), and PHGG-treated and H1N1-infected mice (**d**) at 12 weeks of age. Scale bars, 100 µm. (**e**) Length of ileal villi determined from histological sections. Data are shown for five mice per group, with the bars indicating mean values. ** *p* < 0.01 versus control. (**f**–**k**) Abundance of IFN-γ, IL-1β, IL-6, IL-10, MCP-1, and TNF-α mRNAs normalized by that of GAPDH mRNA, respectively, in the ileum of mice of the four experimental groups at 12 weeks of age. Data are expressed relative to the value for the control group and are means ± SD (*n* = 6 to 8 mice per group). ** *p* < 0.01 versus control; † *p* < 0.05 versus H1N1.

We next investigated the effect of PHGG administration on the intestinal environment of H1N1-infected mice. Villi of the ileum appeared damaged in H1N1-infected mice and were shorter in these animals than in control mice, whereas PHGG treatment protected against such atrophy of intestinal villi (Figure 2a–e). We also examined inflammation-related gene expression in the ileum with RT and real-time PCR analysis. The amounts of interferon-γ (IFN-γ), IL-1β, IL-6, IL-10, monocyte chemoattractant protein-1 (MCP-1), and tumor necrosis factor-α (TNF-α) mRNAs were all increased by H1N1 infection, and these effects, with the exception of that on TNF-α mRNA, were significantly attenuated via PHGG administration (Figure 2f–k).

**Figure 3:**
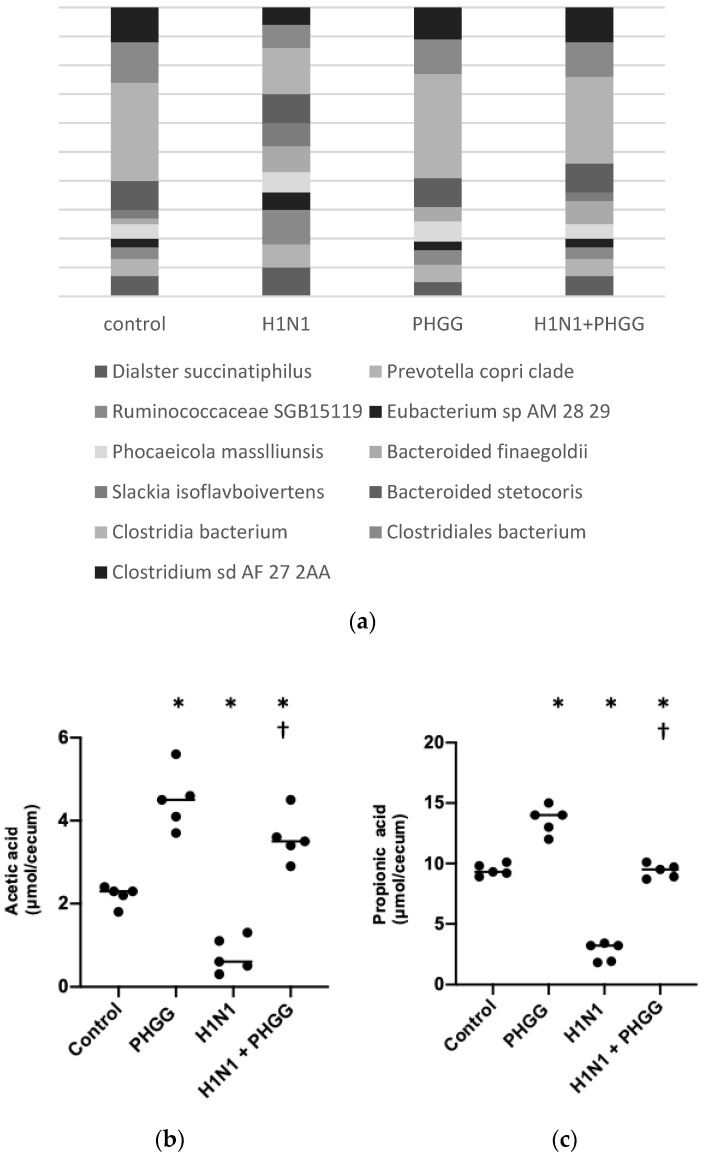

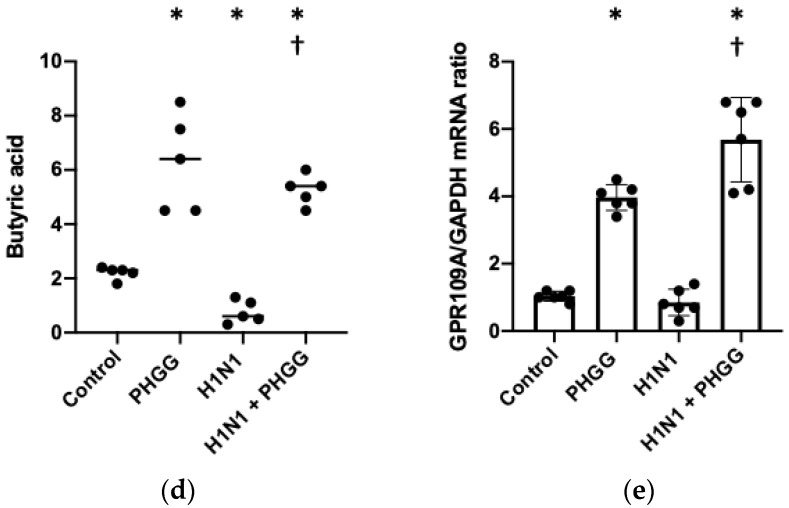
PHGG increased *Clostridia bacterium* in the microbiome (**a**) and SCFA production in the intestine of mice. (**b**–**d**). The amounts of acetic acid (**a**), propionic acid (**b**), and butyric acid (**c**) in {cecal content (30 mg) of mice of the four experimental groups at 12 weeks of age. Data are shown for five mice per group, with the bars indicating mean values. * *p* < 0.05 versus control; † *p* < 0.05 versus H1N1. (**e**) Abundance of GPR109A mRNA normalized by that of GAPDH mRNA in the ileum of mice of the four experimental groups at 12 weeks of age. Data are expressed relative to the value for the control group and are means ± SD (*n* = 6 mice per group). * *p* < 0.05 versus control; † *p* < 0.05 versus H1N1.

We next examined if PHGG altered the gut microbiome and production of SCFAs. Bacterial community composition in mice was monitored via 16S rRNA gene amplicon sequencing. As shown in Figure 3a, *Clostridia* and *Bacteroidia* were the most abundant classes during the experiment in each group. The proportion of *Clostridia* in mice was ~48% with/without PHGG and was ~18% decreased in mice with H1N1 infection. PHGG treatment ameliorated the decrease in *Clostridia* in mice with H1N1 infection (Figure 3a). Given that PHGG has been shown to promote the production of SCFAs in the colon, we measured the levels of acetic acid, propionic acid, and butyric acid in the cecal contents of the study mice. The levels of all three SCFAs were increased by PHGG administration and reduced by H1N1 infection, with the result being that the effects of H1N1 infection were abolished via PHGG administration (Figure 3a–c). SCFAs act as ligands for G protein-coupled receptors, with GPR109A having been found to specifically recognize butyrate [29]. We found that the abundance of GPR109A mRNA in the ileum was increased through PHGG administration in mice with or without H1N1 infection (Figure 3d).

### 3.3. PHGG Inhibited Increases in IgA Production and Serum Cytokine Levels Induced by H1N1 Infection

**Figure 4:**
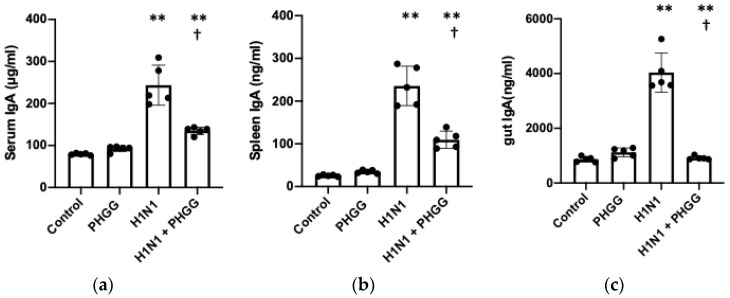

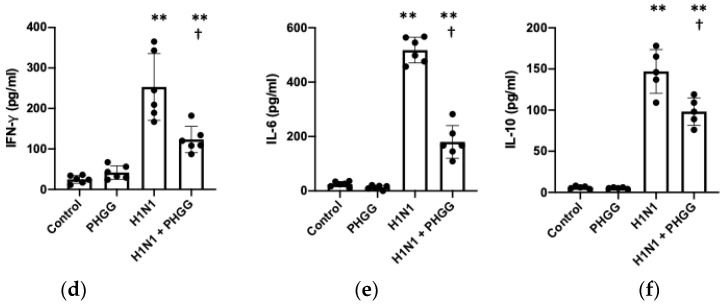
Inhibition by PHGG of the H1N1-induced increases in IgA production and serum inflammatory cytokine levels. (**a**–**c**) IgA concentrations in serum (**a**), spleen homogenate (**b**), and intestinal lavage fluid (**c**) of mice of the four experimental groups at 12 weeks of age. Data are shown for five mice per group, with the bars indicating mean values. ** *p* < 0.01 versus control; † *p* < 0.05 versus H1N1. (**d**–**f**) Serum concentrations of IFN-γ (**d**), IL-6 (**e**), and IL-10 (**f**) in mice of the four experimental groups at 12 weeks of age. Data are shown for five or six mice per group, with the bars indicating mean values. ** *p* < 0.01 versus control; † *p* < 0.05 versus H1N1.

We next focused on systemic levels of IgA, which targets pathogens including viruses on the surface of mucous membranes and thereby limits infection. The serum concentration of IgA was increased by H1N1 infection, and this effect was significantly attenuated by PHGG administration (Figure 4a). The spleen content of IgA was also increased after H1N1 infection in a manner sensitive to inhibition by PHGG (Figure 4b). The concentration of IgA in intestinal lavage fluid showed a small but significant increase in response to PHGG administration alone, whereas the large increase in intestinal IgA level induced by virus infection was prevented by PHGG treatment (Figure 4c). In addition, we measured the serum concentrations of IFN-γ, IL-6, and IL-10, and we found that the levels of all three inflammatory cytokines were increased significantly by H1N1 infection and that PHGG administration inhibited these effects (Figure 4d–f).

### 3.4. Effects of PHGG on Lung Cytokine Gene Expression and Splenocyte Function

**Figure 5:**
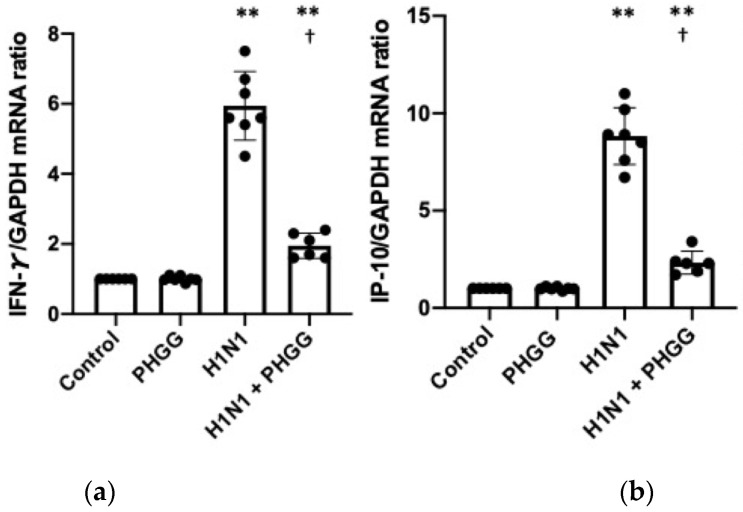

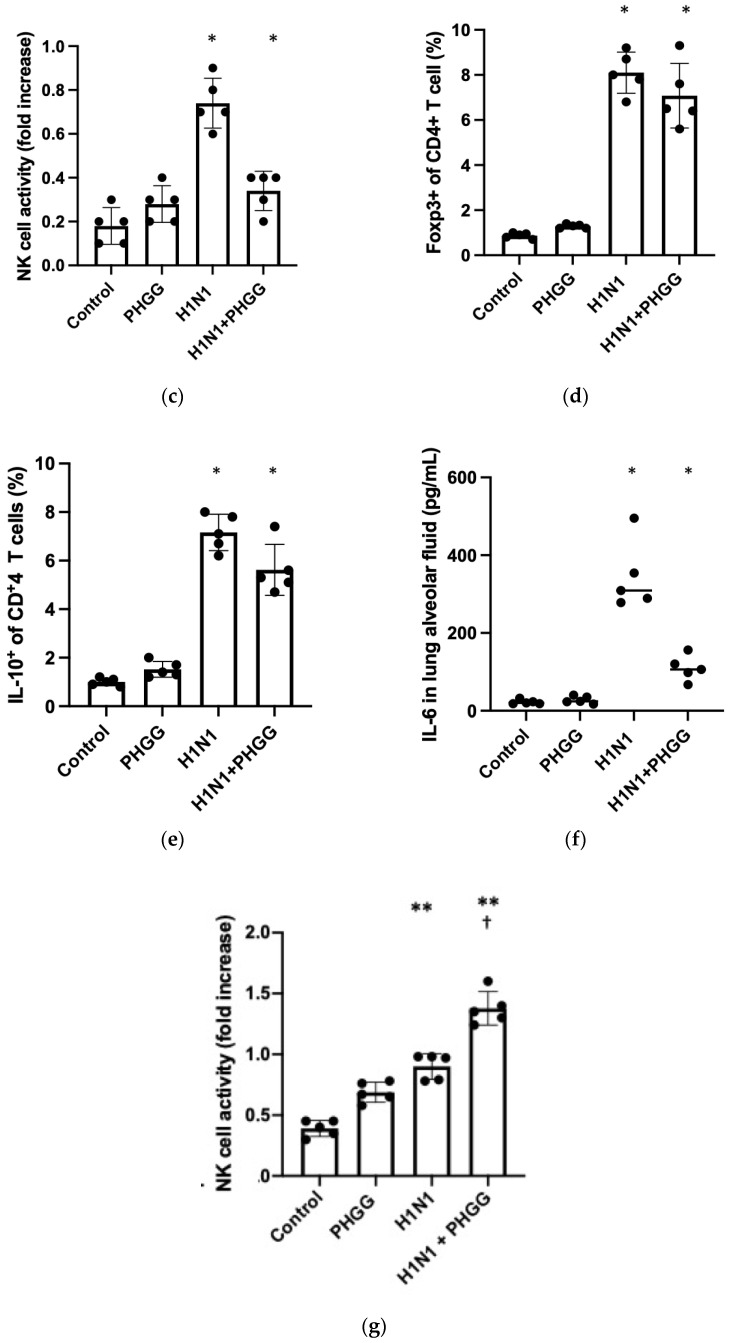

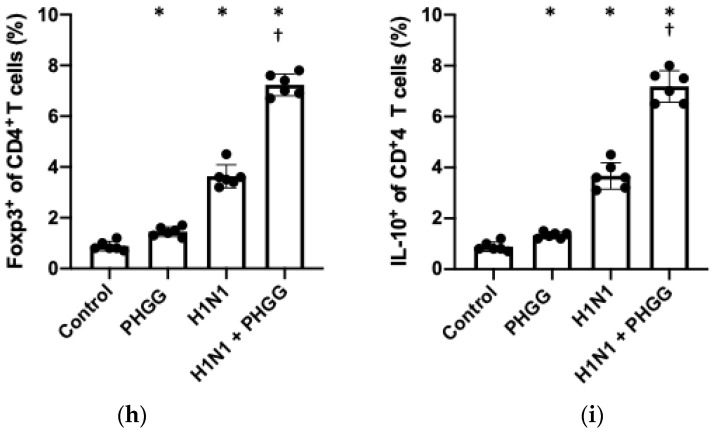
Effects of PHGG administration and H1N1 infection on the immune response in the lung and spleen. (**a**,**b**) Abundance of IFN-γ (**a**) and IP-10 (**b**) mRNAs (normalized by that of GAPDH mRNA) in the lung of mice of the four experimental groups at 12 weeks of age. Data are expressed relative to the value for the control group and are shown for six or seven mice per group, with the bars indicating mean values. ** *p* < 0.01 versus control; † *p* < 0.05 versus H1N1. (**c**) NK cell activity among splenocytes of mice of the four experimental groups at 12 weeks of age. (**d**,**e**) Proportions of Foxp3^+^ (**d**) or IL-10^+^ (**e**) cells among splenic CD4^+^ cells of mice of the four experimental groups at 12 weeks of age. (**f**) IL-6 concentrations in lung alveolar fluid at 12 weeks of age. (**g**) NK cell activity among splenocytes of mice of the four experimental groups at 12 weeks of age. Data are expressed as fold increase and are means ± SD (*n* = 5 mice per group). * *p* < 0.05 versus control; † *p* < 0.05 versus H1N1. (**h,i**) Proportions of Foxp3^+^ (**d**) or IL-10^+^ (**e**) cells among splenic CD4^+^ cells of mice of the four experimental groups at 12 weeks of age. Data are shown for six mice per group together with the mean ± SD (**h**) or mean (**i**) values. * *p* < 0.05 versus control.

Finally, we examined further whether PHGG administration might affect the immune response to H1N1 influenza virus infection in the lung and spleen. The amounts of IFN-γ and IFN-γ-inducible protein-10 (IP-10) mRNAs in lung were greatly increased by H1N1 infection, whereas these increases were markedly attenuated by PHGG administration (Figure 5a,b). In addition, NK cell activity in the lung increased in mice with intake of PHGG (Figure 5c). Flow cytometric analysis also revealed that the proportions of Foxp3^+^ cells and IL-10^+^ cells among splenic CD4^+^ T cells were similarly increased by PHGG administration (Figure 5d,e). Moreover, IL-6 was also ameliorated by PHGG administration in lung alveolar fluid (Figure 5f).

Examination of NK cell activity among splenocytes revealed that both dietary intake of PHGG and H1N1 infection alone increased NK cell activity compared with that in control mice and that the effects of PHGG and H1N1 appeared additive (Figure 5g). To clarify the mechanism of enhanced immune response by PHGG, we examined the association of Foxp3 and IL-10 in the spleen. We also determined that the proportions of Foxp3^+^ cells and IL-10^+^ cells among splenic CD4^+^ T cells were similarly increased by both PHGG administration and H1N1 infection (Figure 5h,i).

## 4. Discussion

**Figure 6:**
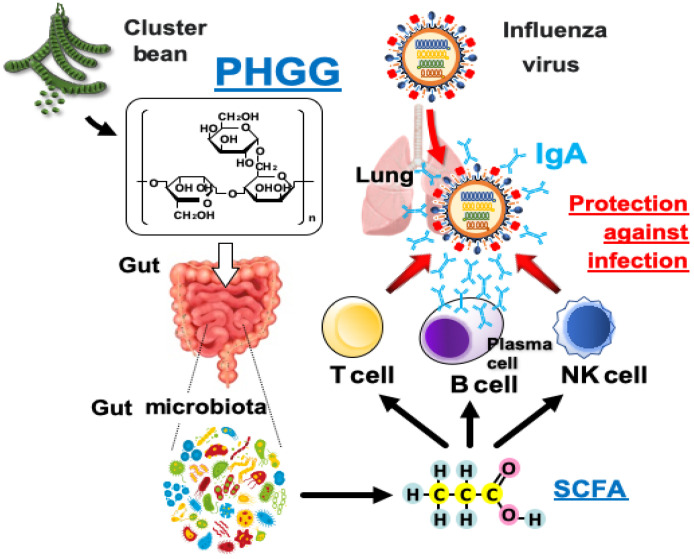
Mechanism of PHGG action on ameliorated influenza virus infection.

In this study, it was shown that PHGG, partially hydrolyzed guar gum, suppresses the aggravation of influenza. PHGG induced an increase in SCFA production, enhanced the immune response as an action of SCFAs, and can thus be considered as effective in suppressing the aggravation of infection (Figure 6). Moreover, it appeard that the SCFAs produced by PHGG induced an increase in Foxp3^+^ cells and IL-10^+^ cells among splenic CD4^+^ T cells. In fact, the effect was observed not only in intestinal immunity but also in the serum cytokine and IgA levels in the spleen and the whole body, and these anti-inflammatory effects resulted in suppression of inflammation in the lung.

Prebiotics, including dietary fiber, have been reported to have a beneficial effect on the gut microbiota and improve the intestinal environment; PHGG is not degraded by digestive enzymes in the digestive tract and is selectively utilized and fermented by beneficial gut bacteria [30]. These bacteria produce SCFAs such as acetoacetic acid, propionic acid, and butyric acid, which regulate pH and thereby further stimulate the growth of beneficial bacteria, forming a positive feedback loop and producing more SCFAs in the gut [30]. PHGG products are fermented by intestinal bacteria in the intestine to produce SCFAs; in particular, butyric acid is produced more than other SCFAs, such as acetic acid and propionic acid [31]. SCFAs can protect or restore the body after inflammation by promoting the induction of M2 macrophages via SCFA receptors and inhibiting neutrophil differentiation [32]. In current study, PHGG potentiated host immunity by promoting the production of IgA- and CD8-positive cells. Affecting regulatory T cells by PHGG is suggested to inhibit excessive immune responses. Several publications have shown that among the SCFAs, butyric acid can ameliorate influenza infection [32,33]. Administration of PHGG is known to be increased by intestinal *clostridium bacteria* [24], and DAT (desaminotyrosine), a metabolite from *clostridium orbiscindens*, increases resistance to infection by the influenza virus via IFN-I signal [34].

Intake of a high fiber diet in mice increases the activity of intestinal bacteria and increases butyric acid production in the cecal content and colonic tissues compared to mice fed a low fiber diet [35]. In addition, butyrate induces the differentiation of colonic Treg cells in mice and enhances the expression of the Foxo3 gene, which is important for inducing differentiation into regulatory T cells. Our study also indicated that this Treg-Foxop3 mechanism is induced by PHGG in mice and enhanced immune response. Moreover, interleukin (IL)-10 production by Treg cells is required for containment of the inflammatory responses in mucosal tissues including the colon [36], and the colonization with a mixture of *Clostridiales* is sufficient to induce IL-10-producing Tregs [37]. Likewise, the intake of PHGG can significantly increase IL-10-producing Treg cells in the spleen. In the present study, we also determined that PHGG intake increased *Clostridium* and subdued inflammation and allergic reactions via activation of regulatory T cells that suppressed immunity in the colon. Furusawa et al. demonstrated that feeding a fiber-rich diet to mice with only *Clostridia* in their intestine increased the number of regulatory T cells and improved symptoms in a mouse model of colitis, Foxop3 gene expression, and activation of regulatory T cells [35]. Taken together, these observations illustrate that butyrate produced by PHGG has a key role in the commensal-microbe-mediated differentiation of functional Treg cells.

The neutralizing effect of IgA on the influenza virus is well known, and it is possible that an increase in IgA inhibits the establishment of influenza virus infection [38]. Secretory IgA prevents mucosally transmitted pathogens and commensal bacteria from binding to epithelial cells and neutralizes their toxins to maintain homeostasis at mucosal surfaces [39]. In the intestinal mucosa, it is known that B cells differentiate into intestinal mucosal lymphatic tissues such as Peyer’s patches and mesenteric lymph nodes and then finally migrate to the intestinal lamina propria and differentiate into IgA-producing plasma cells. Even in the absence of infection, a large amount of IgA is constantly produced. This IgA protects the mucous membrane from innumerable indigenous bacteria and helps maintain the balance of indigenous bacteria. Recent studies suggest that dendritic cells, the control tower of the immune response, play an important role in the constitutive production of IgA [40]. According to Tezuka et al., indigenous bacteria first begin stimulation in the intestine, IFNγ is produced, and the stimulated dendritic cells change to the mucosal type, plasmacytoid cells [39]. In plasmacytoid dendritic cells, a large amount of proteins such as APRIL and BAFF that promote IgA production are expressed, and IgA production is efficiently induced. Since it has been reported that excessive production of APLIL and BAFF is involved in the induction of autoimmune diseases and cancer [41], it is not possible to target these proteins and their producing cells, dendritic cells. Therefore, butyric acids are expected to be a new target for inflammation and viral infections.

In the current study, suppression of intestinal villus atrophy by PHGG administration was observed. A previous report has shown that pectin, a highly fermentable dietary fiber, increases the number of mucosal cells in the lower intestinal tract [42]. Moreover, a study with rats comparing those with and without supplementation of 1.5% PHGG to enteral formula showed that PHGG improved villous atrophy. Studies of butyric acid administered to the cecum of pigs have reported an increase in the number of mucosal epithelial cells [43]. We speculate that the suppression of intestinal villus atrophy by ingestion of PHGG in this study worked through butyric acid, and the improvement of intestinal villus atrophy might contribute to maintaining body weight through the protection of intestinal function. This can be expected to have a preventive effect on bacterial translocation in which intestinal bacteria migrate into the blood.

Our study focused on the role of gut bacterial signaling in modulating the immune response to influenza infection. Epithelial and immune cells of the airways and alveoli play a major role in lung barrier function [44]. In addition to functioning as a physical barrier, epithelial cells secrete mucus and antimicrobial peptides into the airways as a biological defense mechanism, suppressing infection [45]. Many interacting systems, including the autonomic nervous system, are involved in the regulation of immune activation in the lungs [46]. The intestinal environment, represented by the gut microbiota and its metabolites, may influence the immune response in the lungs through this gut–lung axis [47,48]. Most common probiotic strains that have been reported to have a protective effect against influenza infection belong to the *Lactobacillus* and *Bifidobacterium* genera and include *Lactobacillus gasseri* [49], *Lactobacillus plantarum* [50], and *Bifidobacterium longum* [51]. Several probiotic strains have anti-inflammatory activity and exert protective effects against influenza infection in mice. Consistent with many studies, PHGG also exhibited modulation of the anti-inflammatory activity that regulates T cells. In conclusion, we demonstrated that PHGG exerted prebiotic activity by increasing the production of SCFAs, thereby alleviating symptoms of influenza virus infection.

## Figures and Tables

**Table 1:** PCR primer sequences.

Gene	Primer	Sequence (5′-3′)
GAPDH	Forward	TGTCCGTCGTGGATCTGAC
	Reverse	CCTGCTTCACCACCTTCTTG
IFN-γ	Forward	TACTGCCACGGCACAGTCATTGAAA
	Reverse	GCTCTGCAGGATTTTCATGTCACCA
IL-6	Forward	CCTCTCTGCAAGAGACTTCCATCCA
	Reverse	AGCCTCCGACTTGTGAAGTGGT
TNF-α	Forward	CAGGCGGTGCCTATGTCTC
	Reverse	CGATCACCCCGAAGTTCAAGTAG
MCP-1	Forward	CTGTTCACAGTTGCCGGCTG
	Reverse	AGCTTCTTTGGGACACCTGCT
IL-10	Forward	TTTGAATTCCCTGGGTGAGAA
	Reverse	GCTCCACTGCCTTGCTCTTATT
IL-1-β	Forward	TGTTCTTTGAAGTTGACGGACCCC
	Reverse	TGATGTGCTGCTGCGAGATTTGA
IP-10	Forward	TGAGGGCCATAGGGAAGCTTGAAAT
	Reverse	TCCGGATTCAGACATCTCTGCTCAT

**Table 2:** Tissue weights of mice in the four experimental groups at 2 weeks after H1N1 infection.

	Control	PHGG	H1N1	H1N1 + PHGG
Body weight (g)	28.1 ± 0.8	28.6 ± 0.9	22.6 ± 1.2	25.2 ± 0.5
Liver (mg)	1232 ± 45.6	1256 ± 38.6	1431 ± 56.4	1278 ± 47.5
Soleus (mg)	22.3 ± 1.6	22.4 ± 1.8	18.4 ± 1.9	20.5 ± 2.5
EDL (mg)	10.2 ± 0.7	10.1 ± 0.6	8.7 ± 0.9	9.8 ± 0.8
Gastrocnemius (mg)	154.2 ± 10.3	153.8 ± 9.5	121.4 ± 11.1	140.6 ± 10.5
Visceral fat (mg)	45.8 ± 6.7	28.9 ± 5.5	21.2 ± 9.7	22.7 ± 8.7
Subcutaneous fat (mg)	87.3 ± 10.2	70.3 ± 8.9	30.7 ± 12.9	54.2 ± 8.6
Spleen (mg)	89.3 ± 2.3	85.8 ± 1.8	98.1 ± 1.4	90.2 ± 1.8

## Data Availability

The authors confirm that the data supporting the findings of this study are available within the article.
